# Supplementary material for: Pyruvate Dehydrogenase Kinase 1 inhibition mediated oxidative phosphorylation enhancement in cartilage promotes osteoarthritis progression
Source: BMC Musculoskelet Disord. 2023 Jul 20;24:597. doi: 10.1186/s12891-023-06585-6 (PMC10357736; doi:10.1186/s12891-023-06585-6)
Supplement: Supplementary file 1 — Additional file 1. [file 12891_2023_6585_MOESM1_ESM.zip › KEGG.Gsea.1653622432464/gsea_report_for_normal_1653622432464.html]

Report for normal 1653622432464 [GSEA]

| GS  follow link to MSigDB | GS DETAILS | SIZE | ES | NES | NOM p-val | FDR q-val | FWER p-val | RANK AT MAX | LEADING EDGE || 1 | KEGG\_RIBOSOME | Details ... | 84 | -0.66 | -2.78 | 0.000 | 0.000 | 0.000 | 2701 | tags=63%, list=21%, signal=79% |
| 2 | KEGG\_SMALL\_CELL\_LUNG\_CANCER | Details ... | 82 | -0.52 | -2.17 | 0.000 | 0.000 | 0.001 | 1946 | tags=32%, list=15%, signal=37% |
| 3 | KEGG\_DORSO\_VENTRAL\_AXIS\_FORMATION | Details ... | 19 | -0.61 | -1.89 | 0.004 | 0.014 | 0.045 | 3107 | tags=63%, list=24%, signal=83% |
| 4 | KEGG\_PROSTATE\_CANCER | Details ... | 87 | -0.42 | -1.76 | 0.002 | 0.049 | 0.191 | 2021 | tags=29%, list=16%, signal=34% |
| 5 | KEGG\_ADHERENS\_JUNCTION | Details ... | 72 | -0.43 | -1.74 | 0.000 | 0.049 | 0.231 | 2810 | tags=36%, list=22%, signal=46% |
| 6 | KEGG\_JAK\_STAT\_SIGNALING\_PATHWAY | Details ... | 136 | -0.35 | -1.61 | 0.002 | 0.147 | 0.620 | 2367 | tags=27%, list=18%, signal=33% |
| 7 | KEGG\_APOPTOSIS | Details ... | 84 | -0.37 | -1.59 | 0.006 | 0.160 | 0.694 | 2847 | tags=32%, list=22%, signal=41% |
| 8 | KEGG\_ADIPOCYTOKINE\_SIGNALING\_PATHWAY | Details ... | 64 | -0.39 | -1.57 | 0.008 | 0.168 | 0.755 | 2398 | tags=36%, list=19%, signal=44% |
| 9 | KEGG\_FOCAL\_ADHESION | Details ... | 191 | -0.32 | -1.51 | 0.002 | 0.235 | 0.885 | 2926 | tags=30%, list=23%, signal=38% |
| 10 | KEGG\_MAPK\_SIGNALING\_PATHWAY | Details ... | 248 | -0.31 | -1.50 | 0.001 | 0.221 | 0.899 | 2970 | tags=30%, list=23%, signal=38% |
| 11 | KEGG\_ACUTE\_MYELOID\_LEUKEMIA | Details ... | 57 | -0.38 | -1.50 | 0.017 | 0.203 | 0.901 | 2970 | tags=32%, list=23%, signal=41% |
| 12 | KEGG\_PATHWAYS\_IN\_CANCER | Details ... | 310 | -0.29 | -1.49 | 0.001 | 0.213 | 0.937 | 2842 | tags=28%, list=22%, signal=35% |
| 13 | KEGG\_RIG\_I\_LIKE\_RECEPTOR\_SIGNALING\_PATHWAY | Details ... | 63 | -0.37 | -1.49 | 0.024 | 0.197 | 0.937 | 2101 | tags=27%, list=16%, signal=32% |
| 14 | KEGG\_VEGF\_SIGNALING\_PATHWAY | Details ... | 70 | -0.37 | -1.48 | 0.020 | 0.192 | 0.945 | 3057 | tags=34%, list=24%, signal=45% |
| 15 | KEGG\_MTOR\_SIGNALING\_PATHWAY | Details ... | 47 | -0.39 | -1.46 | 0.042 | 0.205 | 0.967 | 2084 | tags=30%, list=16%, signal=35% |
| 16 | KEGG\_BLADDER\_CANCER | Details ... | 42 | -0.40 | -1.46 | 0.055 | 0.197 | 0.968 | 793 | tags=19%, list=6%, signal=20% |
| 17 | KEGG\_PATHOGENIC\_ESCHERICHIA\_COLI\_INFECTION | Details ... | 52 | -0.37 | -1.44 | 0.047 | 0.218 | 0.979 | 2431 | tags=33%, list=19%, signal=40% |
| 18 | KEGG\_INSULIN\_SIGNALING\_PATHWAY | Details ... | 129 | -0.32 | -1.43 | 0.011 | 0.219 | 0.986 | 3423 | tags=40%, list=27%, signal=53% |
| 19 | KEGG\_ENDOMETRIAL\_CANCER | Details ... | 51 | -0.37 | -1.43 | 0.038 | 0.208 | 0.986 | 2705 | tags=33%, list=21%, signal=42% |
| 20 | KEGG\_T\_CELL\_RECEPTOR\_SIGNALING\_PATHWAY | Details ... | 105 | -0.32 | -1.42 | 0.017 | 0.214 | 0.989 | 2970 | tags=30%, list=23%, signal=39% |
| 21 | KEGG\_RENAL\_CELL\_CARCINOMA | Details ... | 70 | -0.35 | -1.42 | 0.030 | 0.205 | 0.989 | 4229 | tags=44%, list=33%, signal=66% |
| 22 | KEGG\_ECM\_RECEPTOR\_INTERACTION | Details ... | 80 | -0.34 | -1.42 | 0.015 | 0.197 | 0.989 | 3790 | tags=43%, list=29%, signal=60% |
| 23 | KEGG\_TIGHT\_JUNCTION | Details ... | 118 | -0.32 | -1.41 | 0.018 | 0.207 | 0.994 | 2507 | tags=24%, list=19%, signal=29% |
| 24 | KEGG\_HEDGEHOG\_SIGNALING\_PATHWAY | Details ... | 45 | -0.36 | -1.37 | 0.067 | 0.254 | 0.998 | 2881 | tags=42%, list=22%, signal=54% |
| 25 | KEGG\_NEUROACTIVE\_LIGAND\_RECEPTOR\_INTERACTION | Details ... | 248 | -0.28 | -1.36 | 0.010 | 0.252 | 0.999 | 3933 | tags=36%, list=30%, signal=51% |
| 26 | KEGG\_TGF\_BETA\_SIGNALING\_PATHWAY | Details ... | 81 | -0.33 | -1.35 | 0.052 | 0.259 | 0.999 | 3140 | tags=36%, list=24%, signal=47% |
| 27 | KEGG\_GNRH\_SIGNALING\_PATHWAY | Details ... | 93 | -0.31 | -1.34 | 0.064 | 0.269 | 0.999 | 3287 | tags=34%, list=25%, signal=46% |
| 28 | KEGG\_GLYCEROPHOSPHOLIPID\_METABOLISM | Details ... | 64 | -0.33 | -1.33 | 0.070 | 0.278 | 0.999 | 2996 | tags=31%, list=23%, signal=41% |
| 29 | KEGG\_CHRONIC\_MYELOID\_LEUKEMIA | Details ... | 72 | -0.33 | -1.33 | 0.084 | 0.274 | 0.999 | 4991 | tags=50%, list=39%, signal=81% |
| 30 | KEGG\_SPLICEOSOME | Details ... | 110 | -0.30 | -1.32 | 0.039 | 0.271 | 0.999 | 2630 | tags=31%, list=20%, signal=38% |
| 31 | KEGG\_NEUROTROPHIN\_SIGNALING\_PATHWAY | Details ... | 120 | -0.30 | -1.32 | 0.052 | 0.263 | 0.999 | 2358 | tags=26%, list=18%, signal=31% |
| 32 | KEGG\_NOD\_LIKE\_RECEPTOR\_SIGNALING\_PATHWAY | Details ... | 56 | -0.34 | -1.32 | 0.082 | 0.256 | 0.999 | 1949 | tags=27%, list=15%, signal=31% |
| 33 | KEGG\_GRAFT\_VERSUS\_HOST\_DISEASE | Details ... | 35 | -0.36 | -1.30 | 0.125 | 0.280 | 0.999 | 1243 | tags=20%, list=10%, signal=22% |
| 34 | KEGG\_THYROID\_CANCER | Details ... | 29 | -0.38 | -1.29 | 0.138 | 0.307 | 1.000 | 3605 | tags=34%, list=28%, signal=48% |
| 35 | KEGG\_AXON\_GUIDANCE | Details ... | 113 | -0.29 | -1.29 | 0.086 | 0.298 | 1.000 | 3361 | tags=34%, list=26%, signal=45% |
| 36 | KEGG\_LEUKOCYTE\_TRANSENDOTHELIAL\_MIGRATION | Details ... | 107 | -0.29 | -1.28 | 0.091 | 0.303 | 1.000 | 4320 | tags=42%, list=33%, signal=63% |
| 37 | KEGG\_LINOLEIC\_ACID\_METABOLISM | Details ... | 25 | -0.40 | -1.28 | 0.159 | 0.297 | 1.000 | 4670 | tags=60%, list=36%, signal=94% |
| 38 | KEGG\_CELL\_ADHESION\_MOLECULES\_CAMS | Details ... | 118 | -0.28 | -1.27 | 0.068 | 0.299 | 1.000 | 4093 | tags=37%, list=32%, signal=54% |
| 39 | KEGG\_NOTCH\_SIGNALING\_PATHWAY | Details ... | 39 | -0.35 | -1.26 | 0.152 | 0.305 | 1.000 | 3543 | tags=41%, list=27%, signal=56% |
| 40 | KEGG\_P53\_SIGNALING\_PATHWAY | Details ... | 61 | -0.31 | -1.26 | 0.116 | 0.300 | 1.000 | 2818 | tags=28%, list=22%, signal=35% |
| 41 | KEGG\_TYPE\_II\_DIABETES\_MELLITUS | Details ... | 44 | -0.34 | -1.25 | 0.135 | 0.312 | 1.000 | 3203 | tags=36%, list=25%, signal=48% |
| 42 | KEGG\_CYTOSOLIC\_DNA\_SENSING\_PATHWAY | Details ... | 45 | -0.33 | -1.24 | 0.161 | 0.322 | 1.000 | 2101 | tags=29%, list=16%, signal=34% |
| 43 | KEGG\_GLIOMA | Details ... | 62 | -0.31 | -1.23 | 0.147 | 0.342 | 1.000 | 1066 | tags=16%, list=8%, signal=17% |
| 44 | KEGG\_WNT\_SIGNALING\_PATHWAY | Details ... | 134 | -0.27 | -1.23 | 0.105 | 0.336 | 1.000 | 2567 | tags=29%, list=20%, signal=36% |
| 45 | KEGG\_ERBB\_SIGNALING\_PATHWAY | Details ... | 83 | -0.29 | -1.21 | 0.159 | 0.353 | 1.000 | 4154 | tags=42%, list=32%, signal=62% |
| 46 | KEGG\_B\_CELL\_RECEPTOR\_SIGNALING\_PATHWAY | Details ... | 71 | -0.30 | -1.21 | 0.140 | 0.347 | 1.000 | 2358 | tags=25%, list=18%, signal=31% |
| 47 | KEGG\_AMYOTROPHIC\_LATERAL\_SCLEROSIS\_ALS | Details ... | 49 | -0.32 | -1.21 | 0.194 | 0.345 | 1.000 | 1979 | tags=24%, list=15%, signal=29% |
| 48 | KEGG\_TOLL\_LIKE\_RECEPTOR\_SIGNALING\_PATHWAY | Details ... | 95 | -0.27 | -1.17 | 0.184 | 0.414 | 1.000 | 2262 | tags=24%, list=18%, signal=29% |
| 49 | KEGG\_ALDOSTERONE\_REGULATED\_SODIUM\_REABSORPTION | Details ... | 39 | -0.32 | -1.16 | 0.245 | 0.434 | 1.000 | 2358 | tags=28%, list=18%, signal=34% |
| 50 | KEGG\_ENDOCYTOSIS | Details ... | 163 | -0.25 | -1.15 | 0.179 | 0.445 | 1.000 | 3537 | tags=34%, list=27%, signal=47% |
| 51 | KEGG\_CHEMOKINE\_SIGNALING\_PATHWAY | Details ... | 169 | -0.24 | -1.14 | 0.197 | 0.462 | 1.000 | 3548 | tags=30%, list=28%, signal=40% |
| 52 | KEGG\_ANTIGEN\_PROCESSING\_AND\_PRESENTATION | Details ... | 75 | -0.26 | -1.10 | 0.274 | 0.538 | 1.000 | 3207 | tags=28%, list=25%, signal=37% |
| 53 | KEGG\_LONG\_TERM\_POTENTIATION | Details ... | 67 | -0.27 | -1.10 | 0.301 | 0.533 | 1.000 | 2766 | tags=30%, list=21%, signal=38% |
| 54 | KEGG\_NON\_SMALL\_CELL\_LUNG\_CANCER | Details ... | 53 | -0.28 | -1.10 | 0.302 | 0.527 | 1.000 | 2358 | tags=23%, list=18%, signal=28% |
| 55 | KEGG\_OLFACTORY\_TRANSDUCTION | Details ... | 66 | -0.27 | -1.09 | 0.285 | 0.533 | 1.000 | 2896 | tags=29%, list=22%, signal=37% |
| 56 | KEGG\_REGULATION\_OF\_ACTIN\_CYTOSKELETON | Details ... | 194 | -0.23 | -1.09 | 0.268 | 0.530 | 1.000 | 3846 | tags=36%, list=30%, signal=51% |
| 57 | KEGG\_PROGESTERONE\_MEDIATED\_OOCYTE\_MATURATION | Details ... | 75 | -0.26 | -1.09 | 0.315 | 0.523 | 1.000 | 4949 | tags=55%, list=38%, signal=88% |
| 58 | KEGG\_PRION\_DISEASES | Details ... | 34 | -0.31 | -1.07 | 0.374 | 0.569 | 1.000 | 2970 | tags=35%, list=23%, signal=46% |
| 59 | KEGG\_CYTOKINE\_CYTOKINE\_RECEPTOR\_INTERACTION | Details ... | 233 | -0.22 | -1.06 | 0.330 | 0.588 | 1.000 | 3700 | tags=32%, list=29%, signal=44% |
| 60 | KEGG\_COLORECTAL\_CANCER | Details ... | 61 | -0.27 | -1.06 | 0.367 | 0.579 | 1.000 | 2705 | tags=25%, list=21%, signal=31% |
| 61 | KEGG\_PHOSPHATIDYLINOSITOL\_SIGNALING\_SYSTEM | Details ... | 72 | -0.26 | -1.05 | 0.369 | 0.590 | 1.000 | 2512 | tags=24%, list=19%, signal=29% |
| 62 | KEGG\_CALCIUM\_SIGNALING\_PATHWAY | Details ... | 166 | -0.22 | -1.05 | 0.361 | 0.581 | 1.000 | 3035 | tags=27%, list=24%, signal=35% |
| 63 | KEGG\_BASAL\_TRANSCRIPTION\_FACTORS | Details ... | 32 | -0.30 | -1.05 | 0.392 | 0.578 | 1.000 | 901 | tags=16%, list=7%, signal=17% |
| 64 | KEGG\_NITROGEN\_METABOLISM | Details ... | 22 | -0.33 | -1.04 | 0.394 | 0.586 | 1.000 | 3881 | tags=41%, list=30%, signal=58% |
| 65 | KEGG\_ARRHYTHMOGENIC\_RIGHT\_VENTRICULAR\_CARDIOMYOPATHY\_ARVC | Details ... | 67 | -0.25 | -1.02 | 0.431 | 0.620 | 1.000 | 3904 | tags=39%, list=30%, signal=55% |
| 66 | KEGG\_PANCREATIC\_CANCER | Details ... | 70 | -0.25 | -1.02 | 0.434 | 0.623 | 1.000 | 1946 | tags=17%, list=15%, signal=20% |
| 67 | KEGG\_MELANOMA | Details ... | 68 | -0.25 | -1.01 | 0.436 | 0.622 | 1.000 | 3209 | tags=28%, list=25%, signal=37% |
| 68 | KEGG\_NATURAL\_KILLER\_CELL\_MEDIATED\_CYTOTOXICITY | Details ... | 117 | -0.22 | -0.99 | 0.468 | 0.675 | 1.000 | 3580 | tags=30%, list=28%, signal=41% |
| 69 | KEGG\_LONG\_TERM\_DEPRESSION | Details ... | 66 | -0.24 | -0.96 | 0.531 | 0.736 | 1.000 | 2970 | tags=23%, list=23%, signal=29% |
| 70 | KEGG\_INOSITOL\_PHOSPHATE\_METABOLISM | Details ... | 49 | -0.25 | -0.96 | 0.530 | 0.727 | 1.000 | 2452 | tags=20%, list=19%, signal=25% |
| 71 | KEGG\_ALANINE\_ASPARTATE\_AND\_GLUTAMATE\_METABOLISM | Details ... | 27 | -0.29 | -0.96 | 0.547 | 0.722 | 1.000 | 1368 | tags=22%, list=11%, signal=25% |
| 72 | KEGG\_LYSINE\_DEGRADATION | Details ... | 38 | -0.26 | -0.95 | 0.531 | 0.721 | 1.000 | 2268 | tags=18%, list=18%, signal=22% |
| 73 | KEGG\_BASAL\_CELL\_CARCINOMA | Details ... | 46 | -0.25 | -0.93 | 0.576 | 0.780 | 1.000 | 2791 | tags=26%, list=22%, signal=33% |
| 74 | KEGG\_FC\_EPSILON\_RI\_SIGNALING\_PATHWAY | Details ... | 75 | -0.22 | -0.91 | 0.645 | 0.820 | 1.000 | 3203 | tags=28%, list=25%, signal=37% |
| 75 | KEGG\_LEISHMANIA\_INFECTION | Details ... | 69 | -0.22 | -0.89 | 0.660 | 0.841 | 1.000 | 1625 | tags=14%, list=13%, signal=16% |
| 76 | KEGG\_TYPE\_I\_DIABETES\_MELLITUS | Details ... | 39 | -0.25 | -0.88 | 0.688 | 0.853 | 1.000 | 2190 | tags=21%, list=17%, signal=25% |
| 77 | KEGG\_ALLOGRAFT\_REJECTION | Details ... | 33 | -0.25 | -0.87 | 0.666 | 0.860 | 1.000 | 400 | tags=9%, list=3%, signal=9% |
| 78 | KEGG\_GAP\_JUNCTION | Details ... | 85 | -0.20 | -0.86 | 0.738 | 0.871 | 1.000 | 2512 | tags=21%, list=19%, signal=26% |
| 79 | KEGG\_VASCULAR\_SMOOTH\_MUSCLE\_CONTRACTION | Details ... | 103 | -0.20 | -0.85 | 0.754 | 0.884 | 1.000 | 3318 | tags=27%, list=26%, signal=36% |
| 80 | KEGG\_VIRAL\_MYOCARDITIS | Details ... | 66 | -0.21 | -0.84 | 0.761 | 0.903 | 1.000 | 780 | tags=11%, list=6%, signal=11% |
| 81 | KEGG\_FC\_GAMMA\_R\_MEDIATED\_PHAGOCYTOSIS | Details ... | 91 | -0.20 | -0.84 | 0.805 | 0.900 | 1.000 | 3380 | tags=30%, list=26%, signal=40% |
| 82 | KEGG\_MELANOGENESIS | Details ... | 90 | -0.19 | -0.79 | 0.871 | 0.972 | 1.000 | 2436 | tags=21%, list=19%, signal=26% |
| 83 | KEGG\_UBIQUITIN\_MEDIATED\_PROTEOLYSIS | Details ... | 116 | -0.18 | -0.78 | 0.905 | 0.973 | 1.000 | 1664 | tags=15%, list=13%, signal=17% |
| 84 | KEGG\_HEMATOPOIETIC\_CELL\_LINEAGE | Details ... | 84 | -0.18 | -0.77 | 0.894 | 0.977 | 1.000 | 1526 | tags=14%, list=12%, signal=16% |
| 85 | KEGG\_ABC\_TRANSPORTERS | Details ... | 36 | -0.22 | -0.77 | 0.846 | 0.971 | 1.000 | 4250 | tags=39%, list=33%, signal=58% |
| 86 | KEGG\_AUTOIMMUNE\_THYROID\_DISEASE | Details ... | 44 | -0.21 | -0.77 | 0.859 | 0.966 | 1.000 | 400 | tags=7%, list=3%, signal=7% |
| 87 | KEGG\_HYPERTROPHIC\_CARDIOMYOPATHY\_HCM | Details ... | 75 | -0.18 | -0.72 | 0.945 | 1.000 | 1.000 | 710 | tags=9%, list=6%, signal=10% |
| 88 | KEGG\_TASTE\_TRANSDUCTION | Details ... | 32 | -0.21 | -0.71 | 0.868 | 1.000 | 1.000 | 4411 | tags=38%, list=34%, signal=57% |
| 89 | KEGG\_GLYCOSPHINGOLIPID\_BIOSYNTHESIS\_LACTO\_AND\_NEOLACTO\_SERIES | Details ... | 24 | -0.21 | -0.68 | 0.897 | 1.000 | 1.000 | 3469 | tags=42%, list=27%, signal=57% |
| 90 | KEGG\_ETHER\_LIPID\_METABOLISM | Details ... | 27 | -0.19 | -0.64 | 0.944 | 1.000 | 1.000 | 3537 | tags=33%, list=27%, signal=46% |
| 91 | KEGG\_ALPHA\_LINOLENIC\_ACID\_METABOLISM | Details ... | 15 | -0.22 | -0.63 | 0.934 | 1.000 | 1.000 | 2750 | tags=27%, list=21%, signal=34% |
| 92 | KEGG\_PPAR\_SIGNALING\_PATHWAY | Details ... | 63 | -0.16 | -0.63 | 0.992 | 1.000 | 1.000 | 2723 | tags=21%, list=21%, signal=26% |
| 93 | KEGG\_ONE\_CARBON\_POOL\_BY\_FOLATE | Details ... | 15 | -0.22 | -0.63 | 0.926 | 1.000 | 1.000 | 3881 | tags=47%, list=30%, signal=67% |
| 94 | KEGG\_COMPLEMENT\_AND\_COAGULATION\_CASCADES | Details ... | 66 | -0.15 | -0.61 | 0.992 | 1.000 | 1.000 | 4455 | tags=35%, list=35%, signal=53% |
| 95 | KEGG\_GALACTOSE\_METABOLISM | Details ... | 24 | -0.17 | -0.54 | 0.986 | 1.000 | 1.000 | 2872 | tags=25%, list=22%, signal=32% |
| 96 | KEGG\_RENIN\_ANGIOTENSIN\_SYSTEM | Details ... | 16 | -0.18 | -0.53 | 0.983 | 0.995 | 1.000 | 4381 | tags=44%, list=34%, signal=66% |
Table: Gene sets enriched in phenotype **normal (10 samples) **[plain text format]****
